# Nucleobindin 2 expression is an independent prognostic factor for bladder cancer

**DOI:** 10.1097/MD.0000000000019597

**Published:** 2020-03-27

**Authors:** Jeong Man Cho, Kyong Tae Moon, Ho Jung Lee, Soon Cheol Shin, Jae Duck Choi, Jeong Yoon Kang, Tag Keun Yoo

**Affiliations:** a Department of Urology, Eulji General Hospital, Eulji University School of Medicine, Seoul, Korea; b Department of Pathology, Eulji General Hospital, Eulji University School of Medicine, Seoul, Korea.

**Keywords:** bladder cancer, nucleobindin 2, urothelial carcinoma

## Abstract

Nucleobindin 2 (NUCB2) has been reported to play an important role in both tumorigenesis and cancer progression. This study aimed to examine the clinical significance of NUCB2 expression urothelial carcinoma of the bladder (UCB).

The expression level of NUCB2 and its correlation with clinicopathological parameters was analyzed in 225 UCB tissues by immunohistochemistry. Kaplan–Meier analysis and Cox proportional hazards regression models were used to investigate the correlation between NUCB2 expression and the prognosis of UCB patients. High NUCB2 expression of UCB patients significantly correlated with aggressive clinicopathological features. Patients with high NUCB2 had shorter overall survival and recurrence-free survival in Kaplan–Meier survival curve (*P* = .018 and *P* = .001, respectively).

Our results show that high expression of NUCB2 associated with aggressive clinicopathological feature and predicted unfavorable prognosis in patients with UCB might serve as feasible biomarker for clinical outcome of UCB patients after surgery and potential therapeutic target in the future.

## 1 Introduction

Bladder cancer (BC) is the most frequently found malignant tumor in the urinary system. It typically appears with higher recurrence and mortality; its postoperative recurrence ratio within 2 years reaches approximately 61% in high-risk BC.^[1]^ Many studies delving into oncogenes for tumorigenesis have been carried out so far. However, the factors that are effective for the prediction of recurrence and prognosis of BC are yet to be clarified.^[2–4]^

Nucleobindin 2 (NUCB2), the precursor protein of nesfatin-1, was originally found in the nucleus of the hypothalamus, and has been known to be related to food intake and energetic homeostasis.^[5,6]^ The manifestation of NUCB2 is also found in peripheral tissues such as pancreatic islet, testis, stomach, and adipose tissue.^[7–10]^ According to recent studies, the roles of NUCB2, which is reportedly relevant to several cancers, are still unclear.^[11–16]^ In cases of breast cancer, prostate cancer, clear cell renal cell carcinoma, and endometrial carcinoma, the manifestation of NUCB2 has been known to be related to bad consequences.^[11,16–18]^ However, the manifestation NUCB2 and its roles in cases of BC are yet to be reported. Thus, the present study intends for an identification of the manifestation of NUCB2 in BC and relevance of the manifestation of NUCB2 to clinicopathological characteristics to determine whether NUCB2 could be a prognostic factor predicting the recurrence of BC.

## 2 Materials and methods

The subjects of the present study were 225 patients diagnosed with BC who underwent operations (transurethral BC resection, partial excision of bladder, or radical cystectomy) in our hospital during the period from 2004 to 2014. All cases were identified as bladder urothelial carcinoma by a pathologist. The pathological data were classified according to the 2010 Union for International Cancer Control (UICC) TNM classification for the stage and 2004 WHO classification for the grade. The medical records of subjects were collected and the demographics, histologic grade, pathological stage, tumor size, presence of solitary or multiple lesions, recurrence, and overall survival (OS) were analyzed. The OS was calculated by using the date of last follow-up or death of each patient.

In order to analyze NUCB2 protein expression, paraffin-embedded specimens were cut into 3-μm thick sections for immunohistochemical staining. After baking at 65°C for 1 hour, the sections were dewaxed in xylene, hydrated through a graded series of alcohols (100%, 95%, and 85%) and rinsed in deionized water. The sections were then heated in citric acid buffer (pH 6.0), using a microwave oven, for antigen retrieval. To block endogenous peroxidase activity, sections were treated with 3% hydrogen peroxide solution for 15 minutes. The sections were then incubated overnight with primary rabbit polyclonal antibody (Sigma-Aldrich, St Louis, MO) at a 1 : 250 dilution at 4°C in a humidified chamber. The next day, the sections were incubated sequentially with biotinylated goat anti-rabbit secondary antibody for 30 minutes. After the last washing step in phosphate-buffered saline (PBS), the sections were incubated in substrate solution [3,30-diaminobenzidine (DAB)], counterstained in hematoxylin, dehydrated through an alcohol gradient and xylene and mounted.

All samples were analyzed by 2 pathologists. The level of expression of NUCB2 protein was semi-quantitatively classified by a combination of proportion and positive intensity of stained immunoreactive cells.

The percentage of positive tumor cells was scored as follows: 0 (no positive tumor cells), 1 (<10% positive tumor cells), 2 (10–50% positive tumor cells), and 3 (>50% positive tumor cells). The staining intensity was scored as follows: 0 (no staining), 1 (weak staining), 2 (moderate staining), and 3 (strong staining). The sum of the staining intensity score and percentage score was used to define NUCB2 protein expression levels: 0 to 2, low expression and 3 to 4, high expression.

SPSS Version 19.0 (SPSS Inc., Chicago, IL) was used for the statistical analyses of data collected in the present study. A Chi-square test was carried out to determine the correlation between NUCB2 protein expression and clinicopathological characteristics.

OS curves and recurrence-free survival curves were plotted according to the Kaplan–Meier method and subgroups were compared by conducting a log-rank test. A Cox proportional hazards model was adopted for univariate and multivariate analysis in order to identify possible prognostic factors. A *P* value < .05 was considered to be statistically significant. This study was retrospective and did not require IRB approval at the time of the study.

## 3 Results

The 225 subjects consisted of 183 male subjects and 42 female subjects. The age of the subjects ranged from 36 years to 88 years; the average age of all subjects was calculated as 70.3 years. The follow-up period for each patient was distributed in the interval from 3 months to 60 months; the average period of follow-up was calculated as 44.3 months. The clinicopathological features of all subjects are as summarized in Table 1.

**Table 1:**
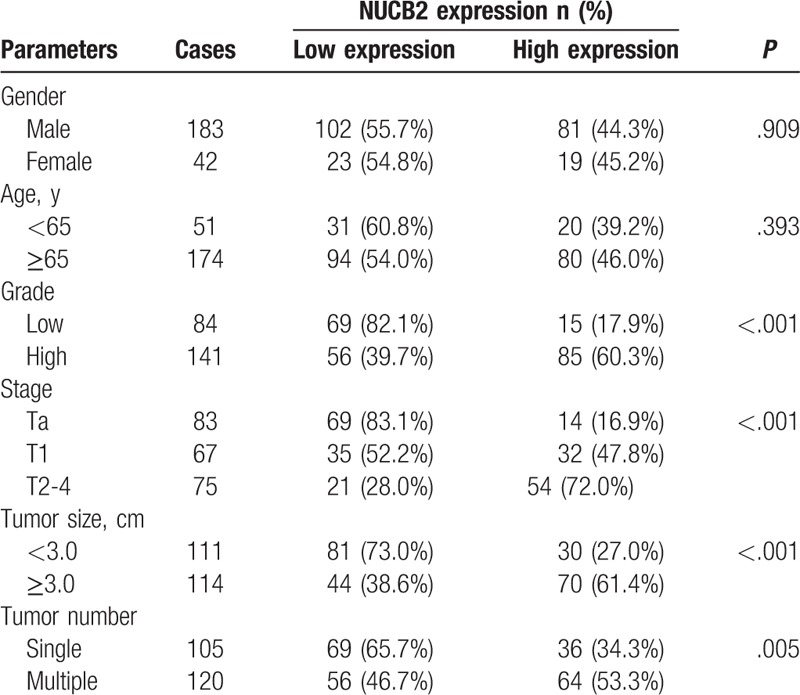
Relationship between NUCB2 expression and clinicopathological features.

Among all 225 BC tissues, 163 tissues (72.4%) manifested NUCB2. The correlation between the manifestation of NUCB2 and clinicopathological features was analyzed, as summarized in Table 1. High expression of NUCB2 appeared relevant to aggressive clinicopathological features such as tumor grade (*P* < .001), TNM stage (*P* < .001), tumor size (*P* < .001), and tumor number (*P* = .005). However, it appeared irrelevant to gender (*P* = .909) and age (*P* = .393). Kaplan-Meier survival curves were plotted to estimate the survival of patients of BC that manifested NUCB2 ().

**Figure 1:**
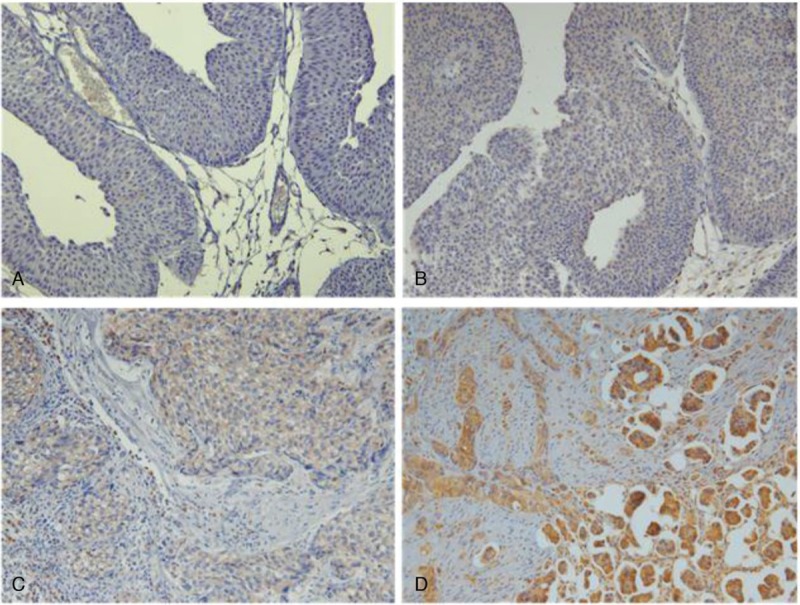
Immunohistochemical expression of NUCB2 in BC tissue. (A) 0 (no positive tumor cells), (B) 1 (<10% positive tumor cells), (C) 2 (10–50% positive tumor cells), and (D) 3 (>50% positive tumor cells).

**Figure 2:**
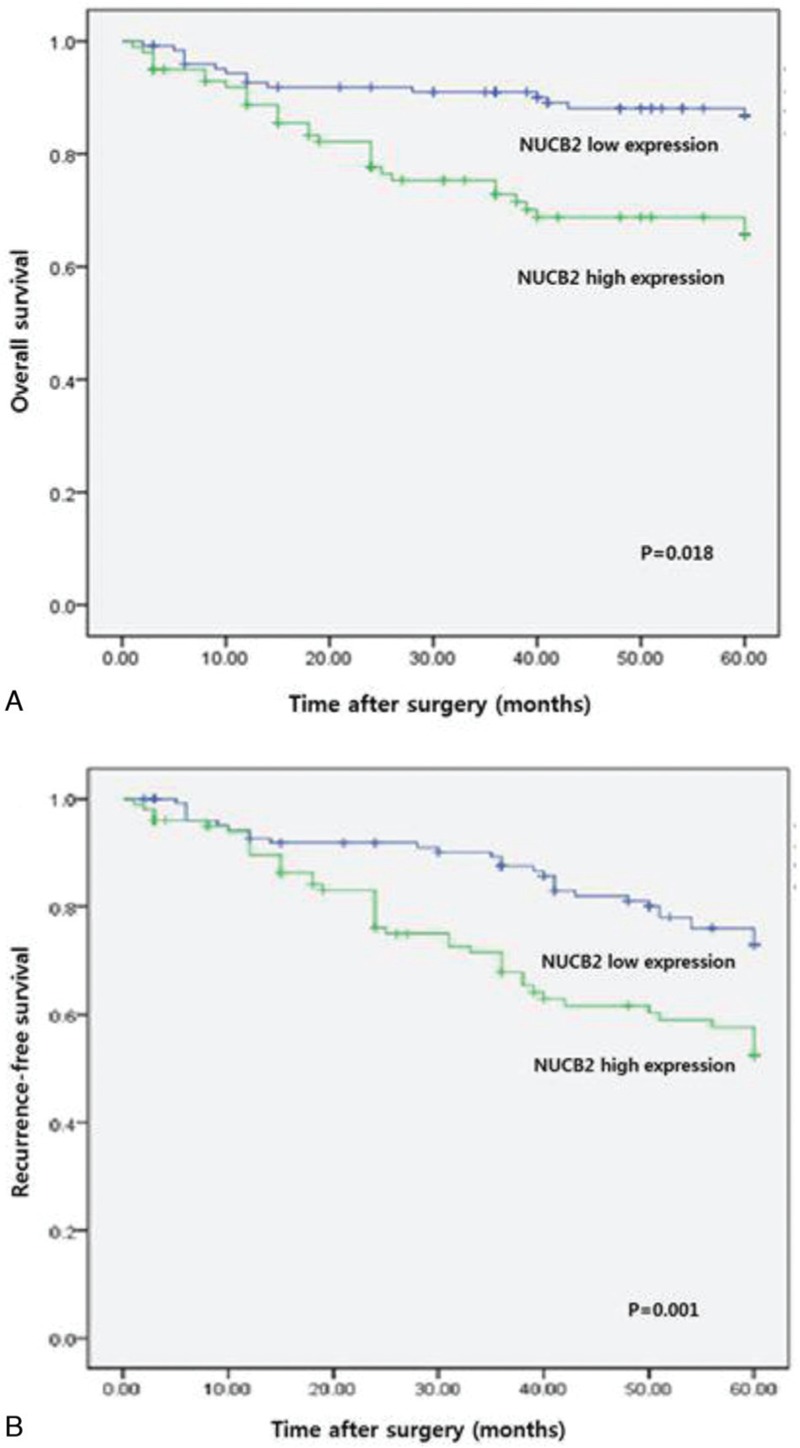
Kaplan–Meier curve for survival in patients with urothelial carcinoma of the bladder (UCB) according to expression level of NUCB2. (A) Overall survival (low expression vs high expression). (B) Recurrence-free survival (low expression vs high expression).

The patients who manifested high expression of NUCB2 showed worse OS and recurrence-free survival than patients who manifested lower expression of NUCB2 (Fig. 2A and B; *P* = .018 and *P* = .001, respectively). Univariate analysis was carried out with the use of a Cox proportional hazard model from which the tumor grade, T stage, tumor size, NUCB2 expression, and tumor number were identified as significant prognostic factors predicting recurrence-free survival. Also, only tumor grade, T stage, and NUCB2 expression were identified as independent prognostic factors predicting the recurrence-free survival of patients in the multivariate analysis (Table 2).

**Table 2:**
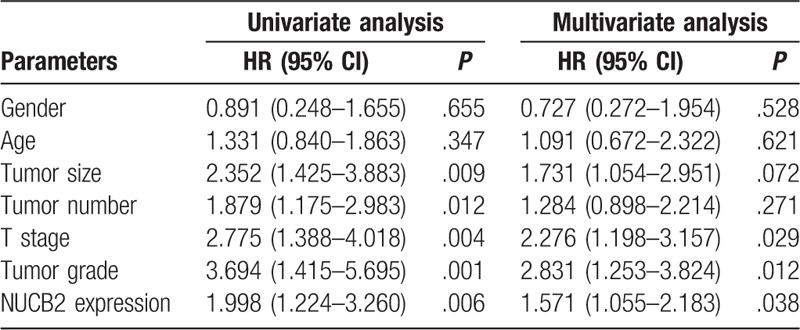
Prognostic parameters for disease-free survival in univariate and multivariate analysis.

## 4 Discussion

Among genitourinary cancers, urothelial cancer of the bladder (UCB) is the most frequently found one. Approximately 70% to 80% of all cases are initially diagnosed as nonmuscle-invasive ones, while the remaining 20% to 30% are diagnosed as muscle-invasive ones. Approximately 30% to 50% of the cases of nonmuscle-invasive BC experience postoperative recurrence, while approximately 10% to 20% of the cases of nonmuscle-invasive BC experience postoperative recurrence of muscle-invasive BC.^[20]^ In recent years, researchers have found that inflammation marker and circulating tumor cells and long noncoding RNAs can influence tumor recurrence and progression.^[21–24]^ In particular, the inflammatory cell markers neutrophil-lymphocyte ratio (NLR) have been used to evaluate cancer recurrence and survival.^[25]^ Also, NLR was a predictor of OS [hazard ratio (HR), 1.19], cancer-specific survival (CSS) (HR, 1.40), recurrence-free survival (RFS) (HR, 1.58), and progression-free survival (PFS) (HR, 1.33) in the most recent meta-analysis, which included 17 studies (only 4 of these studies included patients with NMIBC).^[26]^

According to previously conducted studies, the manifestation of NUCB2 has been associated with bad prognoses in cases of breast cancer, prostate cancer, clear cell renal cell carcinoma, and endometrial carcinoma.^[11,16–18]^ In particular, in an in vitro study of breast cancer, NUCB2 was reported to play important roles in the migration, proliferation, and invasion of BC cells.^[18]^ To our knowledge, this is the first report to investigate the association between NUCB2 and BC.

In the present study, the expression of NUCB2 in BC exhibited an association with aggressive clinicopathological features. High expression of NUCB2 also appeared to be associated with a higher histologic grade and advanced T stage, which are relevant to bad prognoses. Consequently, we thought NUCB2 might be a potential prognostic predictor. In addition, according to results obtained from multivariate analysis, it was concluded that NUCB2 was an OS independent prognostic factor of BC.

The limitation of the present study is the retrospective data collection employed for the retrospective examination. However, despite this limitation, the expression of NUCB2 was concluded to be an independent prognostic factor predicting the consequences for patients suffering from BC. Future prognostic studies should take this molecular marker into consideration and integrate it into established prognostic models.

In conclusion, the high expression of NUCB2 in BC tissues was identified in the present study. The high expression of NUCB2 was found to be associated with aggressive clinicopathological features and bad consequences of the disease. However, further studies are suggested to confirm NUCB2 as a feasible biomarker capable of predicting clinical outcomes of BC treatment.

## 5 Conclusion

The high expression of NUCB2 in BC tissues was identified in the present study. The high expression of NUCB2 was found to be associated with aggressive clinicopathological features and bad consequences of the disease. However, further studies are suggested to confirm NUCB2 as a feasible biomarker capable of predicting clinical outcomes of BC treatment.

## Author contributions

**Conceptualization:** Jeong Man Cho, Tag Keun Yoo.

**Data curation:** Jeong Man Cho, Kyong Tae Moon, Ho Jung Lee.

**Investigation:** Jeong Man Cho.

**Methodology:** Jeong Man Cho, Kyong Tae Moon, Ho Jung Lee, Jae Duck Choi, Tag Keun Yoo.

**Visualization:** Jeong Man Cho, Jae Duck Choi.

**Writing – original draft:** Jeong Man Cho.

**Formal analysis:** Kyong Tae Moon.

**Supervision:** Kyong Tae Moon, Ho Jung Lee, Jae Duck Choi, Jeong Yoon Kang, Tag Keun Yoo.

**Validation:** Kyong Tae Moon, Jae Duck Choi.

**Writing – review & editing:** Jeong Yoon Kang, Tag Keun Yoo.
